# A novel intergenic region (chr2: 30,193,816)-ALK fusion shows sensitivity to Alectinib in lung adenocarcinoma

**DOI:** 10.1186/s12890-023-02351-5

**Published:** 2023-02-08

**Authors:** Ting Wang, Ge Du, Man Niu, Rui Liu

**Affiliations:** 1 Department of Respiratory Medicine, Xi’an People’s Hospital (Xi’an No.4 Hospital), No.21 Jiefang Road, Xi’an, 710004 China; 2 Department of Rehabilitation Center for Elderly, Beijing Rehabilitation Hospital Affiliated to Capital Medical University, Beijing, 100144 China; 3 Department of Pathology Medicine, Xi’an People’s Hospital (Xi’an No.4 Hospital), Xi’an, 710004 China

**Keywords:** Anaplastic lymphoma kinase, Intergenic, Lung cancer, Case report

## Abstract

**Background:**

Anaplastic lymphoma kinase (ALK) rearrangement, which is mostly showed as fused with echinoderm microtubule-associated protein-like 4 gene (EML4), accounts 3–7% of all common mutations in non-small lung cancer (NSCLC). An intergenic region (chr2: 30,193,816), which located on upstream of the adjacent ALK gene, was never been reported as a ALK patterner before.

**Case presentation:**

A 56-year-old female patient who had symptoms of persistent cough and shortness of breath visited our facility on April 24, 2022. The chest computerized tomography (CT) examination revealed a massive right hydrothorax. After draining pleural effusion, a hilar mass accompanied multiple nodules in both lungs could been seen in image. Tracheoscopy revealed neoplasm in the medial segment of the middle lobe of the right lung, and the patient was diagnosed as lung adenocarcinoma pathologically. It tested positive for cytokeratin (CK) 7, NapsinA, ALK, and thyroid transcription factor-1 (TTF-1). Next generation sequence testing confirmed the presence of the intergenic region (chr2: 30,193,816)-ALK fusion in the tumor tissue. The patient was subsequently treated with Alectinib, and her symptoms are obviously relieved, the right hilar mass and metastatic nodule were reduced in the reexamination after three months.

**Conclusions:**

The intergenic region (chr2: 30,193,816)-ALK fusion, which is firstly reported in lung adenocarcinoma, is a mutation with expression significance. It shows sensitivity to Alectinib.

## Background

Lung cancer remains the leading cause of cancer related deaths worldwide [1]. Due to lacks of specific performance, the majority of lung cancer patients are often diagnosed at advanced stage of the disease [2]. Conventional chemotherapy with platinum containing dual drugs was always the first line of treatment for these patients. However, even though this regimen was given at diagnosis, these individuals’ median survival time was less than one year [3]. Along with the driver genes were identified in a subset of NSCLCs, the treatment strategy for this kind of malignancy has changed to more personalized approach based on its molecular markers. For patients with advanced lung cancer harboring activating mutations, targeted therapy has greatly improved their prognosis. Common targets include mutations of epidermal growth factor receptor (EGFR), ALK, proto-oncogene receptor tyrosine kinase (ROS1), etc. [4]. ALK, which is commonly found to be fused to the EML4, accounted for 3–7% of all common mutations in NSCLC [5]. EML4-ALK fusion oncogene (also known as ALK rearrangement) promotes cell proliferation and growth. The ALK Tyrosine kinases inhibitors (TKI) Alectinib has been considered as the first choice for ALK-positive NSCLC [6]. Here, we reported a novel intergenic region (chr2: 30,193,816)-ALK fusion in a female NSCLC case responded well to Alectinib. We retrospectively analyzed the clinical data of this rare case and reviewed the related literature.

## Case presentation

**Fig. 1:**
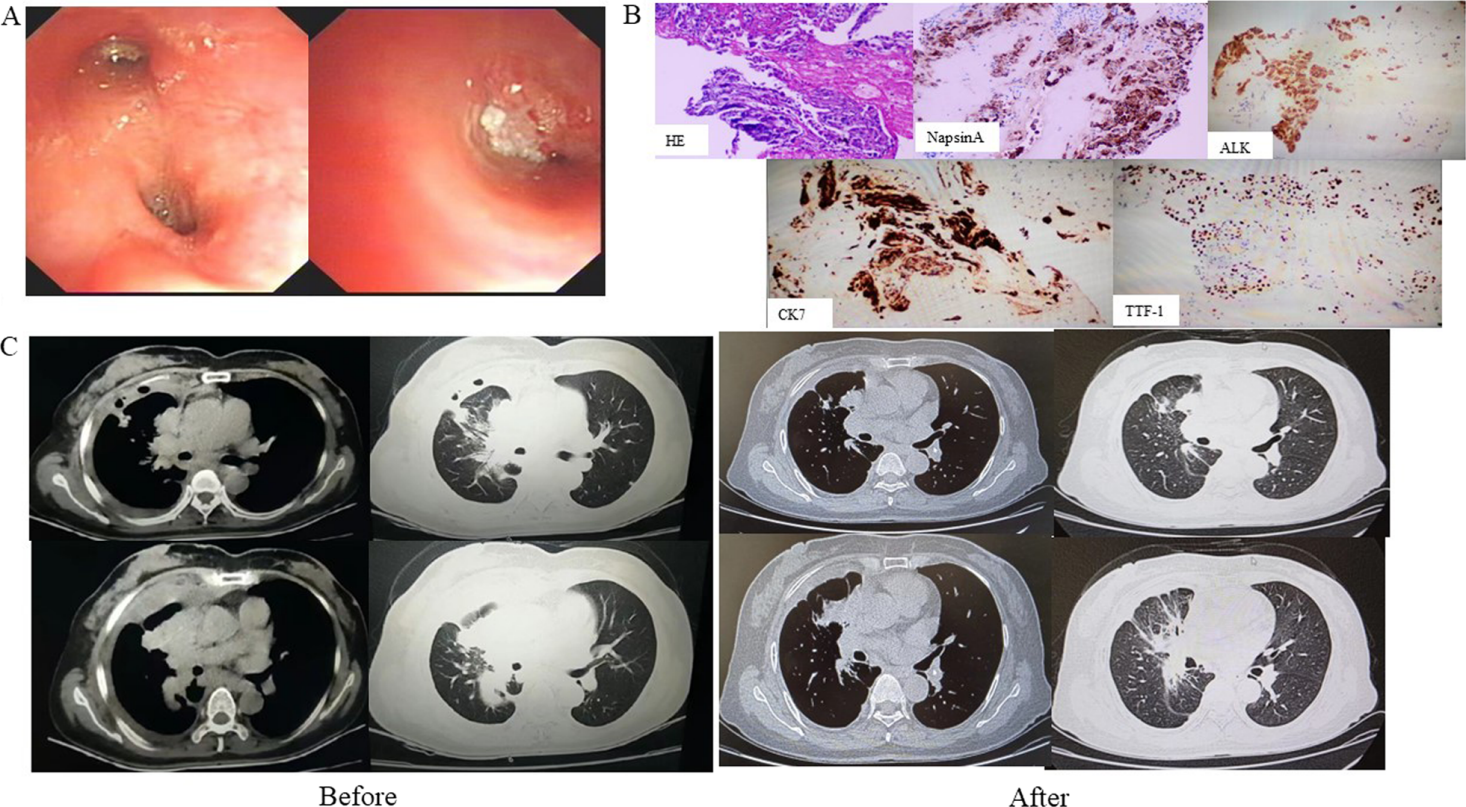
**A** Tracheoscopy revealing neoplasm in the medial segment of the middle lobe of the right lung; **B** IHC analysis indicated the sample being positive for Napsin A, TTF-1, CK7 and ALK, suggesting a primary lung adenocarcinoma (× 400). **C** Radiological features before and after Alectinib treatment

**Fig. 2:**
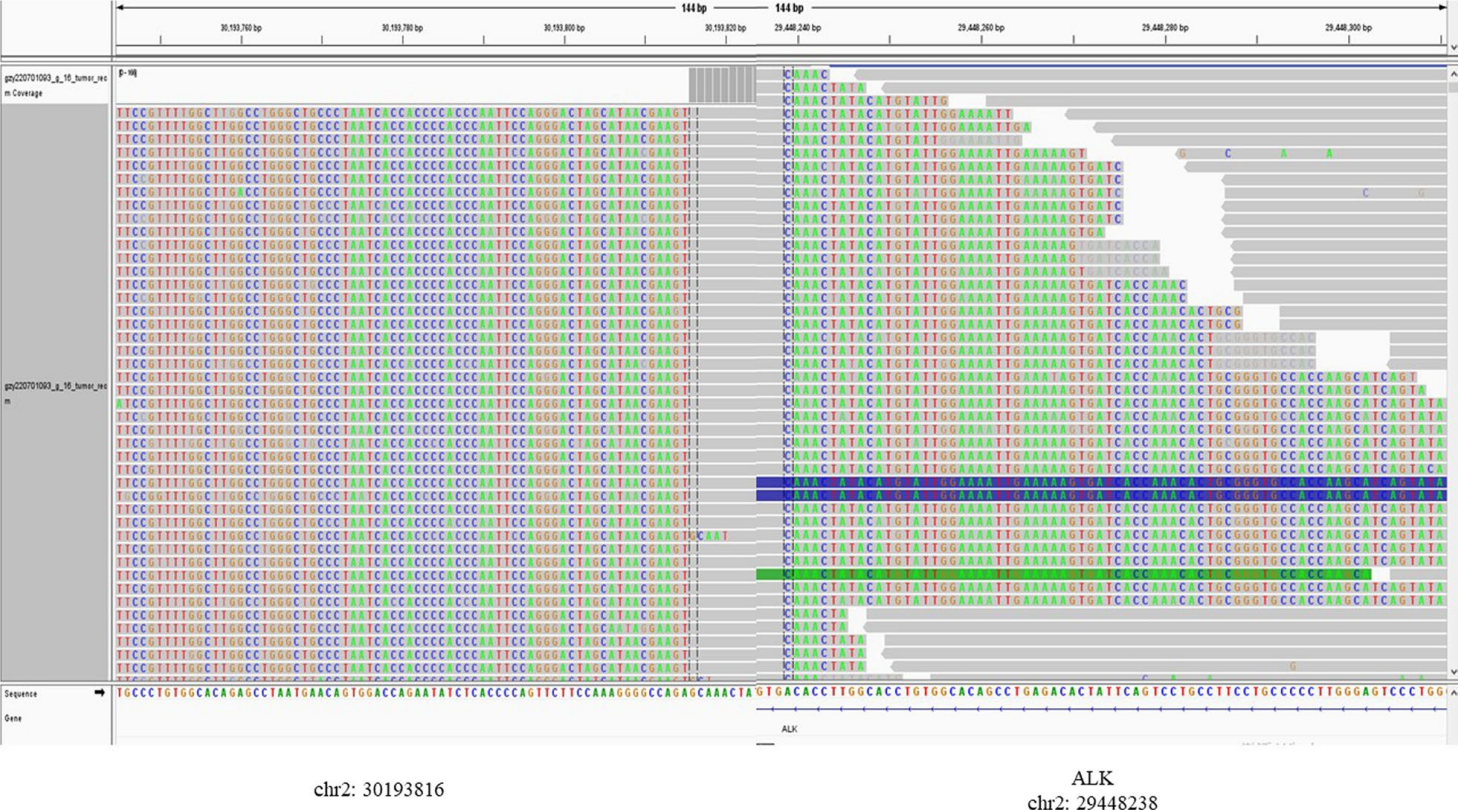
Integrative Genomics Viewer (IGV) visualized the sequencing reads of ALK and intergenic region (chr2: 30,193,816)

**Fig. 3:**
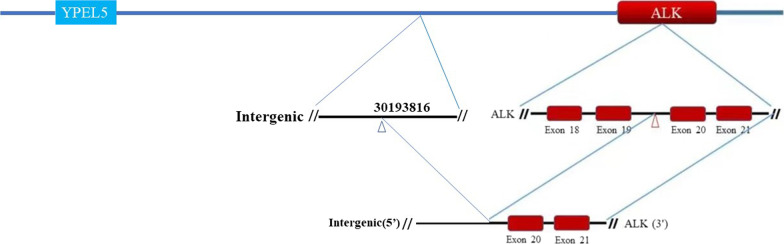
A schematic map showed the structure of the intergenic region (chr2: 30,193,816)-ALK fusion locus

A 56-year-old woman with no history of smoking was admitted to the respiratory department of our hospital on April 24, 2022, with a history of cough and dyspnea over the past six months. Chest computed tomography revealed a massive right hydrothorax. The patient underwent thoracocentesis, and pleural fluid tumor markers, including carcinoembryonic antigen (CEA), cytokeratin 21-1 fragment, and carbohydrate antigen 19-9 (CA199) were significantly elevated. A reexamination of chest CT after pleural effusion drainage demonstrated a hilar mass accompanied multiple nodules in both lungs. Subsequently, tracheoscopy was performed, which unveiled neoplasm in the medial segment of the middle lobe of the right lung (Fig. 1A). The pathological diagnosis was adenocarcinoma, and immunohistochemical analysis showed that the tumor cells were positive for CK7, NapsinA, ALK, and TTF-1 (Fig. 1B). The following next-generation sequencing (NGS) analysis (Yunying Medical Inspection Institute) confirmed that the intergenic region (chr2: 30,193,816)-ALK fusion (with an abundance of 8.10%) was positive (Figs. 2, 3), suggesting may be benefit from the treatment of Alectinib or Crizotinib. We prescribed Alectinib for the patient, and the dose was 600 mg two times per day. Reexamination after three months showed an obvious decrease of the hilar mass and other nodules, no recurrence of pleural effusion (Fig. 1C). The patient had no apparent side effect after therapy but a slight abnormal liver function in first two weeks.

## Discussion and conclusions

ALK, a proto-oncogene encoding anaplastic lymphoma kinase, was primarily found to have an expression in the nervous system [7]. In cancer cells, its signaling could be activated via gene fusion, gene amplification and point mutations, then, signaling cascades, such as mitogen-activated protein kinase (MAPK), (phosphatidylinositol 3-kinase) PI3K/(protein kinase B) AKT, Janus kinase/signal transducer and activator of transcription (JAK/STAT), and mitogen-activated protein kinase kinase 5-extracellular signal-regulated kinase 5 (MEK5-ERK5) pathways could be triggered [8]. In 2007, ALK was first identified in NSCLC by Japanese scholars to have an abnormal fusion with EML4 gene, resulting in activation of downstream pathways and rapid cell proliferation [9]. On the other hand, this fusion severs as a therapeutic target for an ALK‐tyrosine kinase inhibitors, such as Alectinib and Crizotinib. Over the last few years, compared to conventional chemotherapy, ALK inhibitors have shown significant benefits in the treatment of ALK‐positive NSCLC. Alectinib has been recommended by the National Comprehensive Cancer Network (NCCN) guidelines as a first-line treatment for advanced metastatic ALK‐positive NSCLC [10].

At present, in NSCLC, more than 20 fusion partners for ALK have been reported. Mengnan Li et al. performed NGS analysis based on a 168-gene panel on lung adenocarcinoma tissue specimen from 56-year-old Chinese women and detected Huntingtin-interacting protein 1 (HIP1)-ALK fusion [11]. This variant was resistant to Crizotinib while sensitive to Alectinib. Misako Nagasaka et al. reported a 66-year-old nonsmoker man presenting with metastatic adenocarcinoma with striatin (STRN)-ALK fusion revealed a positive response to Alectinib [12]. However, Yoko Nakanishi et al. reported a case of a 51-year-old man who was diagnosed as lung adenocarcinoma harboring STRN-ALK fusion unfortunately did not respond to Alectinib [13]. A novel fusion of the intergenic region between LINC00478/LINC01549 and ALK exon 20 in lung adenocarcinoma was firstly reported by Wei Peng et al., and this fusion responded well to Alectinib [14]. In a large cohort of 6576 NSCLC patients, 343(5.2%) cases were identified to harbor ALK rearrangements. Fourteen cases with complex ALK arrangements were further classified into three types base on their genomic features, including intergenic (*n* = 3), intragenic (*n* = 5) and “bridge joint” rearrangements (*n* = 6). Thirteen cases of them were confirmed to have EML4-ALK fusion expression by RNA-based NGS and immunohistochemistry (IHC). Besides, 9 of 11 cases were positive in situ hybridization (FISH) testing. Further data analysis showed that there was no difference in progression-free survival (PFS) between patients with canonical ALK fusions and patients with complex ALK rearrangements [15]. Researchers believe that an integrated application of multiple detection methods, including DNA-based NGS, RNA and protein level assay may be critical in validating the function of complex ALK rearrangements in NSCLC, selecting the optimal treatment decision and evaluating the patient’s prognosis. The best diagnostic approach to the detection of ALK re-arrangement is controversial, and IHC/FISH/RT-PCR/NGS carry its own merits and demerits. Those suspected positive expression markers in IHC report could be confirmed further by FISH. Meanwhile, for those mutations that may be missed by IHC/FISH due to limited material, NGS could be a complementary diagnostic method. Regarding to the mutation from intergenic region, IHC or FISH report should also be referred to confirm it expression at the transcribed level before make the prescription. They complement each other. In our case, ALK was also detected positive in IHC analysis, suggesting this fusion played a meaningful role in regulating the protein expression.

To our knowledge, this is the first report describing the intergenic region (chr2: 30,193,816) and ALK exon 20 in lung adenocarcinoma. The positive response suggested this novel fusion could be a potential Alectinib-sensitive variant. However, it is noteworthy that Chr2: 30,193,816 is located on the region between the ALK and YPEL5 gene. Using GENSCAN (http://hollywood.mit.edu/GENSCAN.html), we found that only the ALK intracellular region remains when protein translation is predicted based on this intergenic region (chr2: 30,193,816)-ALK fusion. The coiled-coil region, which normally forms dimers and trimers, is also absent. Since there have been reports of ALK fusion genes that are not predicted to form a multimer, it is possible that ALK functions as a monomer as a oncoprotein. In future research, we will explore the specific mechanism of this monomer based on this fusion gene.

We also believe our case will provide a better understanding of this rare fusion (ALK and intergenic region gene) in NSCLC and provide a reference for future clinical treatment.

## Data Availability

All the data supporting our findings is contained within the manuscript.

## Abbreviations

ALK: Anaplastic lymphoma kinase
EML4: Echinoderm microtubule-associated protein-like 4 gene
NSCLC: Non-small lung cancer
CT: Computerized tomography
CK: Cytokeratin
TTF-1: Thyroid transcription factor-1
EGFR: Epidermal growth factor receptor
TKI: Tyrosine kinases inhibitors
CEA: Carcinoembryonic antigen
CA199: Carbohydrate antigen 19-9
NGS: Next-generation sequencing
MAPK: Mitogen-activated protein kinase
PI3K: Phosphatidylinositol 3-kinase
AKT: Protein kinase B
JAK/STAT: Janus kinase/signal transducer and activator of transcription
MEK5-ERK5: Mitogen-activated protein kinase kinase 5-extracellular signal-regulated kinase 5
NCCN: National comprehensive cancer network
HIP1: Huntingtin-interacting protein 1
STRN: Striatin
IHC: Immunohistochemistry
FISH: Situ hybridization
PFS: Progression-free survival
RT-PCR: Reverse transcription-polymerase chain reaction
YPEL: Yippee-like
